# Validation of a novel semi-automated ECG quantification tool, applied to a cardio-oncology: Semi-automated ECG Tool applied to cardio-oncology

**DOI:** 10.1186/s40959-025-00405-7

**Published:** 2025-12-19

**Authors:** Samuel D. Cohen, Maxime Robert-Halabi, Adrien Procureur, Mathieu Jamelot, Martino Vaglio, Fabio Badilini, Edi Prifti, Joe-Elie Salem

**Affiliations:** 1 Department of Pharmacology, Sorbonne University, INSERM CIC-1901, Pitié-Salpêtrière Hospital, APHP, 47 Boulevard de L’Hôpital, Paris, 75013 France; 2 IRD, Sorbonne University, UMMISCO, INSERM, NutriOmics, Paris, France; 3 Department of Medical Oncology, Institut Universitaire de Cancérologie, Sorbonne University, AP-HP, Tenon Hospital, Paris, France; 4 AMPS LLC, New York, NY USA; 5 Holy Spirit University of Kaslik (USEK) University, P.O. Box 446, Jounieh, Lebanon

**Keywords:** Methods, Cardio-oncology, QT interval, ECG, Pharmacology

## Abstract

**Background:**

Electrocardiogram (ECG) analysis is crucial to detect cardiotoxicity. Manual methods are time-consuming and limited by inter-reader variability, highlighting the need for precise, reproducible and rapid semi-automated digital tools in clinical practice.

**Objective:**

This study evaluates the triplicate concatenation method (TCM) using a semi-automated ECG software (CalECG-4.2, AMPS®) by assessing intra- and inter-reader variabilities in two distinct cardio-oncology populations: breast cancer patients receiving ribociclib (a QT-prolonging drug) and patients admitted with severe immune checkpoint inhibitors (ICI)-myocarditis, a condition marked by QRS alterations.

**Methods:**

A total of 420 ECG from 31 patients (21 ribociclib, and 10 ICI-myocarditis) were independently analyzed by two readers. Variability was assessed using Bland–Altman analyses and intraclass correlation coefficients (ICC). Linear mixed-effects modelling quantified time-dependent changes in heart rate (HR), PR, QTc (Fridericia’s HR correction), QRS duration and voltage (Sokolow-Lyon) accounting for inter-reader variability.

**Results:**

Intra and inter-reader reproducibility was excellent (ICC > 0.98, including Sokolow-Lyon voltage; standard-deviation < 4 ms across all time-derived parameters). In ribociclib-treated patients (cycles of 21/28 days on drug), QTc peaked at day 14 (16 ± 1 ms, *p* < 0.001) before decreasing by day 28 (-6 ± 1 ms, *p* < 0.001) compared to baseline. In ICI-myocarditis, QRS duration increased at day 5 before returning to baseline starting day 28, while Sokolow-Lyon voltages increased progressively on immunosuppressive treatments, peaking at day 28 (458 ± 49 µV, *p* < 0.001) and remaining constant afterwards for the next month.

**Conclusion:**

TCM with CalECG-4.2 ensures a high reproducibility while monitoring key parameters like QTc duration and Sokolow-Lyon voltage, making it a reliable and time-saving alternative for the ECG surveillance of drug toxicities in cardio-oncology.

**Supplementary Information:**

The online version contains supplementary material available at 10.1186/s40959-025-00405-7.

## Background

The electrocardiogram (ECG) is an essential tool in clinical and research settings to diagnose and monitor cardiac diseases. Cardio-oncology has emerged as a new field specializing in the surveillance and treatment of cardiovascular toxicities caused by various cancer therapies [1–4]. Such treatments include cyclin-dependent kinase inhibitors, like ribociclib, which may lead to life-threatening *torsades de pointes* by prolonging the heart rate corrected QT interval (QTc) [4–6]. On the other hand, immune checkpoint inhibitors (ICI) can induce severe myocarditis, a rare but potentially fatal condition [7–10]. ECG monitoring of such patients revealed several conduction abnormalities, such as PR and QRS prolongation as well as voltage changes [11, 12]. The latter two have been associated with worse outcomes in ICI-myocarditis, as they likely indicate myocardial edema and/or damage from macrophage infiltration [11, 12].

A thorough monitoring of these ECG parameters is thus indicated but may be challenging in clinical practice. While manual measurement methods are widely used, they are time-consuming and prone to inter-reader, intra-reader and beat-to-beat variability, which limits their reliability [13–15]. For instance, QTc measurements can vary due to differences in baseline selection and correction formulas applied, leading to inconsistencies that limit the diagnostic accuracy [14, 15]. Similarly, since the assessment of Sokolow voltage is highly dependent on the precision of manual tracing measurements, it is intrinsically associated to a high degree of variability and poor reliability [16, 17].

Software using automated methods relying on mathematical concepts, such as CalECG, offer a promising alternative by providing consistent and precise measurements, particularly in complex clinical settings [13]. Notwithstanding these improvements, accurate automated ECG measurements may still be hindered by signal noise and artifacts. To address these issues and further enhance the signal-to-noise ratio, triplicate ECG of 10 s and superimposed median beats have been implemented [17, 18]. Semi-automated methods, such as the triplicate assessment method (TAM), are widely adopted. TAM consists of repeating measurements three times (individual analysis of 10 s ECG within a triplicate series) to ensure accuracy and reliability, which, although more precise, can be labor-intensive. The triplicate concatenation method (TCM) has been introduced as a novel and more efficient technique that build a 30-s ECG by concatenating three 10-s tracing while excluding artifacts at the concatenation points [13, 19]. Although promising, this novel method remains to be validated in large real-life patient populations, particularly for automated QRS voltage and Sokolow calculations, which were previously unavailable in readily available ECG analysis software.

Building on prior research validating the CalECG 3.7 software® for QTc measurement [13], this study aims to evaluate the reproducibility of TCM using the updated CalECG 4.2 software®. This updated version integrates a more precise analysis of the QRS segments and its voltage, enabling a semi-automated calculation of the Sokolow-Lyon voltage, a feature previously unavailable. By assessing intra- and inter-reader variabilities as well as characterizing ECG changes over time, we seek to determine whether TCM offers a reliable alternative for ECG interval measurements in cardio-oncology patients. Specifically, we focus on patients treated with ribociclib or followed for immune checkpoint inhibitor (ICI)-induced myocarditis, since these patient populations exhibit greater ECG variability, thereby providing a robust test for this novel method.

## Methods

### Study population

We recruited thirty-one patients followed in a French quaternary care University-affiliated cardio-oncology center (Pitié-Salpêtrière Hospital, Paris) and enrolled in NEOCARDIO (*NCT03882580,* approved by the Ethics Committee of Sorbonne University on March 12th, 2021), an observational, ambispective cohort. They were first seen between December 2022 and July 2024 and then evaluated prospectively. On inclusion, each patient had 3 resting 12-lead 10-s ECG, each separated by 2-min intervals. Patients were placed in supine position and asked to remain immobile during the ECG acquisition to minimize physiological variability and artefacts. Included patients were required to have high-quality non-electro stimulated ECG in sinus rhythm. Included patients were divided into two groups:

1. *Breast cancer patients*: Twenty-one patients undergoing their first cycle of ribociclib. Each patient is followed for three visits: at day 0 (baseline), at days 14 ± 3 and 28 ± 3 (post-treatment). Ribociclib was administered on a 28-day cycle, with 21 days on-treatment followed by 7 days off-treatment, as part of the standard treatment regimen. Our main focus was to evaluate the treatment-induced effect on the QTc interval, as ribociclib is known to prolong it [4, 20].
2. *ICI myocarditis patients*: Ten patients with severe ICI-induced myocarditis were enrolled. Each patient had 8 planned visits within the first two months of their initial admission. The immunosuppressive regimen consisted of abatacept administered by intravenous injection with concomitant oral ruxolitinib and corticosteroids. Their dosing and administration regimen have previously been published [10, 21]. The main focus was to study the evolution of the QRS voltage and duration while on treatment, as these parameters have been shown useful to assess myocardial injury and inflammation and may represent a surrogate marker for disease severity and treatment response [11, 12].

In this ECG software validation study, six key ECG parameters were analyzed: heart rate (HR), PR interval, QRS duration, corrected QT interval for HR using Fridericia's formula (QTc) [22], and Sokolow-Lyon voltage, calculated using the formula: (S wave maximal voltage in leadV1) + (R wave maximal voltage in lead V5 or lead V6) [23].

### Electrocardiogram recording and analysis

**Fig. 1:**
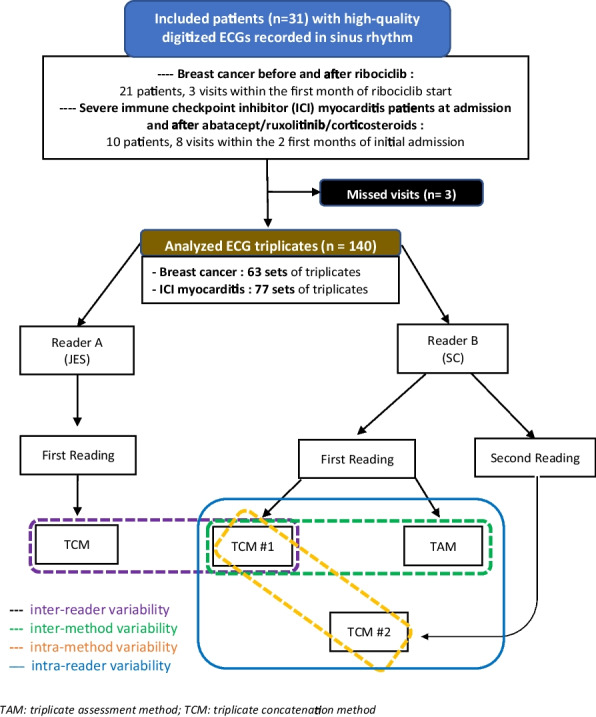
Flow-chart of the study

ECG were recorded using a standardized digital electrocardiograph (ELI 280, V2.4.1.8; Mortara Instrument, Inc., Milwaukee, WI, USA) by trained nurses. The device has a sampling rate of 1000 Hz and a filter of 150 Hz, which were selected to capture detailed signal characteristics for subsequent analysis. In total, 420 ECG (140 triplicate sets) were analyzed (Fig. 1 for detailed flow-chart**)**. Quantitative ECG analysis was performed using the TAM and the TCM, both being semi-automated approaches. Methods variability were assessed by comparing inter-method variability (TAM vs TCM#1 reader B) and intra-method variability (TCM#1 vs TCM#2 reader B). TCM#1 corresponds to the first analysis of the triplicate, while TCM#2 refers to the second analysis. Inter-reader variability for TCM was determined by comparing results between reader A and reader B (Fig. 1). Reader A (JES), a Cardiologist, has over 15 years of experience in ECG analysis, while reader B (SDC), a PharmD, has more than 3 years of experience in ECG analysis. Both readers independently reviewed and analyzed the ECG. They remained blinded to each other's measurements and the study hypotheses throughout the process.

### CalECG 4.2 software® overview

**Fig. 2:**
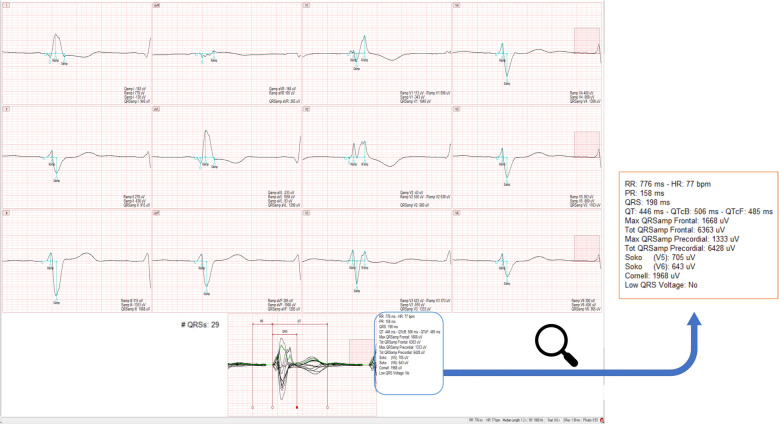
Example of an ECG Analysis in a severe ICI myocarditis patient using TCM with CalECG 4.2®. Superimposed median beat from 29 QRS with vector magnitude (green). Automatic caliper placements (PR, QRS, QT onset, offset and peaks) with manual editing option. QTc (Fridericia’s -QTcF-; and Bazett’s -QTcB- heart rate correction) was derived from the averaged RR intervals. The Sokolow-Lyon (Soko) voltage indices are calculated by measuring the S wave voltage in V1 + the R wave voltage in V5 (Soko V5) or V6 (Soko V6). The pathological QRS duration (> 120 ms) observed herein, reflects the severity of the ICI-myocarditis. Low QRS voltage is coded as "YES" when all frontal leads have a voltage ≤ 500 µV or all precordial leads have a voltage ≤ 1000 µV. Conversely, it is coded as "NO" if any frontal lead exceeds 500 µV or any precordial lead exceeds 1000 µV

All ECG measurements were conducted using the latest version of the CalECG 4.2 software®, which automatically generates a representative beat for each of the 12 leads derived from detected sinus rhythm beats. This process involves aligning sinus rhythm ECG beats on the R-wave’s peak for each lead and creating a median beat by computing a median value for each ECG sample, producing a single representative signal, 1.2 s long, per lead. As a result, the representative beat is not an actual ECG recorded beat but rather an averaged signal, synthesizing the characteristics of all recorded sinus rhythm beats in each lead. To build this representative beat, the software automatically eliminates all premature ventricular contractions within a 10-s ECG recording. The final output includes 12 representative beats, one for each lead. By superimposing these single-lead representative beats, a superimposed median beat is generated. This superimposed median beat is optimally represented by a vector of magnitude, calculated as the square root of the sum of the squared representative beats. The vector of magnitude facilitates automated measurements of PR, QT and QRS intervals and voltages using a threshold-based method. If automatic PR/QT/QRS fiducial marks are misplaced, users retain the ability to manually adjust the fiducial markers for critical points, including the onset of the P-wave, the onset and offset of the QRS complex or the offset of the T-wave. Additional ECG parameters of interest, particularly QRS voltages can be measured using the same process, as illustrated in Fig. 2. Compared with the previous version (CalECG 3.7 software®) [13], the main improvement of the 4.2 version is a comprehensive revision of the AMPS Bravo algorithm. This update specifically targets waveforms within the QRS complex (Q, R, S, R’, S’), which were previously excluded from automated analysis and could only be measured manually in the 3.7 version. The addition of automated QRS voltage analysis, integrating Sokolow-Lyon and microvoltage assessments, enhances measurement precision and reliability.

### Statistical analyses

Reliability between readers and methods was assessed using mixed-effects Bland–Altman models and intraclass correlation coefficients (ICC). ICC were calculated as two-way random-effects, absolute-agreement, single-measure [ICC(2,1)] with 95% confidence intervals, following Koo & Li. Mixed-effects Bland–Altman models included random intercepts for subject and timepoint, and were also used to test for proportional bias (difference regressed on mean) [24, 25]. Bland–Altman plots graphically represent the agreement between the two readers/readings, plotting the mean difference (bias) and the limits of agreement (LOA) (mean difference ± 1.96 standard deviation [SD]) against the average of the two measurements. A non-inferiority (NI) objective for QTc comparing TCM vs TAM was a margin of ± 5 ms and the requirement that the upper bound of the 90% CI was < 10 ms (E14 FDA guidance). Two one-sided tests (TOST) were implemented on paired differences (α = 0.05), with the 90% CI and p-values reported. Repeatability coefficient (RC) was defined as 1.96 × SD of paired within-method differences, computed for all metrics. Linear mixed-effects models (LME, lmer R-package) were fitted with fixed effects for measurement method, reader, reading, visit and demographics, and a random intercept for subject. Multiplicity was addressed by Benjamini–Hochberg false discovery rate, applied across secondary endpoints and time contrasts; the primary endpoint (QTc) was prespecified and reported with unadjusted *p*-values. All statistical analyses were performed using R software (version 4.4.2). A *p*-value of < 0.05 was deemed significant.

## Results

**Fig. 3:**
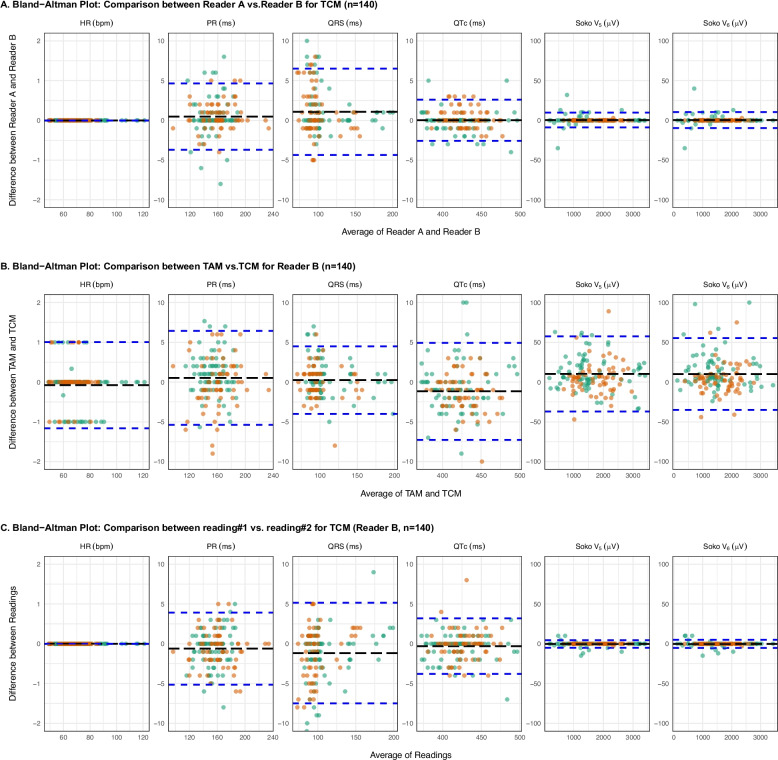
Bland–Altman Manual Plot for all analyses by cohort. The dashed blue lines represent the limits of agreement (LOA), and the dashed black line indicates the mean bias for the different measurements. For the cohorts, the orange points represent breast cancer patients on ribociclib, while the green points represent immune checkpoint inhibitor (ICI) myocarditis patients. Soko for Sokolow-Lyon voltage

The ICC and Bland–Altman analysis (including LOA) for the method/readings/readers comparisons are summarized in Table 1 and Fig. 3.

**Table 1:** Summary of ICC, RC and Bland–Altman Analysis for Method Comparisons. This table displays ICC, RC and Bland–Altman analysis results for three method comparisons assessing specific ECG parameters, including HR, PR, QRS, QTc and Sokolow-Lyon indices (V5 and V6)

Parameters	ICC	95%CI ICC	Mean ± SD Bias	Upper LOA (95% CI)	Lower LOA (95% CI)	RC
TCM 1 reader A vs. TCM 1 reader B (***n*** = 140)						
HR (bpm)	1	[1-1]	0 ± 0	0	0	0
PR (ms)	0.995	[0.992–0.996]	0.47 ± 2.13	4.65	−3.70	5.55
QRS (ms)	0.994	[0.989–0.996]	1.08 ± 2.77	6.51	−4.35	6.95
QTc (ms)	0.999	[0.998–0.999]	0.02 ± 1.32	2.61	−2.57	3.64
Soko V5 (µV)	1	[1-1]	0.40 ± 4.78	9.76	−8.96	13.24
Soko V6 (µV)	1	[1-1]	0.42 ± 5.12	10.45	−9.61	14.18
TAM 1 reader B vs. TCM 1 reader B (***n*** = 140)						
HR (bpm)	0.999	[0.999–0.999]	−0.08 ± 0.55	1.00	−1.16	1.08
PR (ms)	0.989	[0.985–0.992]	0.53 ± 3.02	6.44	−5.39	5.92
QRS (ms)	0.997	[0.995–0.998]	0.24 ± 2.17	4.50	−4.01	4.25
QTc (ms)	0.992	[0.987–0.995]	−1.17 ± 3.12	4.93	−7.28	6.11
Soko V5 (µV)	0.999	[0.999–1]	10.40 ± 24.10	57.60	−36.90	47.20
Soko V6 (µV)	0.999	[0.999–1]	10.10 ± 23.00	55.20	−35.00	45.10
TCM (reading #1) vs. TCM (reading #2) for reader B (***n*** = 140)						
HR (bpm)	1	[1-1]	0 ± 0	0	0	0
PR (ms)	0.993	[0.990–0.995]	−0.61 ± 2.32	3.93	−5.16	4.54
QRS (ms)	0.991	[0.986–0.994]	−1.17 ± 3.23	5.15	−7.50	6.33
QTc (ms)	0.998	[0.997–0.998]	−0.31 ± 1.78	3.19	−3.80	3.49
Soko V5 (µV)	1	[1-1]	−0.29 ± 2.44	4.51	−5.08	4.79
Soko V6 (µV)	1	[1-1]	−0.19 ± 2.60	4.90	−5.28	5.09

*bpm* beats per minute, *CI* Confidence Interval, *HR* Heart rate, *ICC* Intraclass correlation coefficients, *LOA* Limits of agreement, *NA* Not applicable, *RC* Repeatability coefficient, *SD* Standard deviation, *Soko* Sokolow-Lyon voltage

### Measurements variability with CalECG4.2®

Inter-reader variability for TCM measurements demonstrated excellent consistency across measurements for all ECG parameters (ICC > 0.994 [0.989–0.996], see Table 1). For QTc, the mean bias was 0.02 ± 1.32 ms (LOA: −2.57 to 2.61 ms, RC = 3.64). The PR interval exhibited a mean bias of 0.47 ± 2.13 ms (LOA:−3.70 to 4.65 ms, RC = 5.55). The QRS duration showed a mean bias of 1.08 ± 2.77 ms (LOA: −4.35 to 6.51 ms, RC = 6.95). The Sokolow-Lyon voltage using lead V5 demonstrated a mean bias of 0.40 ± 4.78 μV (LOA:−8.96 to 9.76 µV, RC = 13.24).

Inter-method variability between TAM and TCM demonstrated high consistency across measurements for all ECG parameters (ICC > 0.989 [0.985–0.992], see Table 1). For QTc, the mean bias was −1.17 ± 3.12 ms (LOA: −7.28 to 4.93 ms, RC = 6.11), and its 90% CI was − 1.61 to − 0.74 ms, fully within ± 5 ms, thus supporting NI. The PR interval exhibited a mean bias of 0.53 ± 3.02 ms (LOA: −5.39 to 6.44 ms, RC = 5.92). The QRS duration showed a mean bias of 0.24 ± 2.17 ms (LOA: −4.01 to 4.50 ms, RC = 4.25). The Sokolow-Lyon voltage using lead V5 demonstrated a mean bias of 10.40 ± 24.10 μV (LOA: −36.90 to 57.60 µV, RC = 47.20).

Intra-method variability within TCM demonstrated excellent repeatability across measurements for all ECG parameters (ICC > 0.991 [0.986–0.994], see Table 1). For QTc, the mean bias was −0.31 ± 1.78 ms (LOA: −3.80 to 3.19 ms, RC = 3.49). The QRS duration showed a mean bias of −1.17 ± 3.23 ms (LOA: −7.50 to 5.15 ms, RC = 6.33). The Sokolow-Lyon voltage using lead V5 demonstrated a mean bias of −0.29 ± 2.44 μV (LOA: −5.08 to 4.51 µV, RC = 4.79).

### Application in real life cohort: ribociclib-treated breast cancer and ICI myocarditis patients

**Fig. 4:**
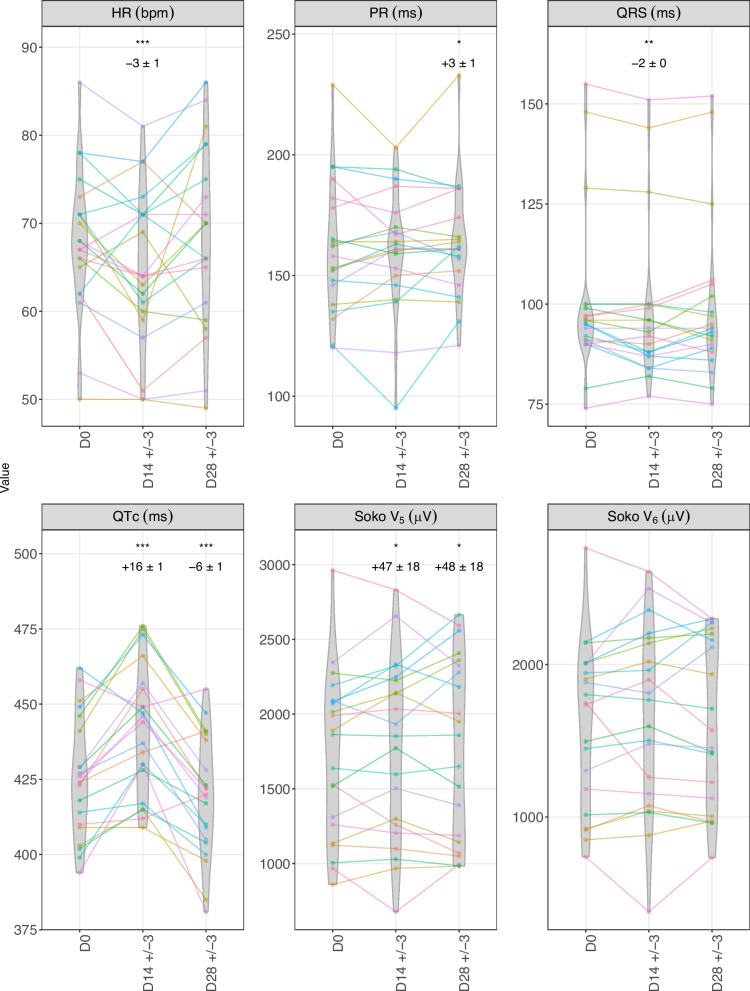
Electrocardiographic changes over time in ribociclib-treated breast cancer patients. Soko for Sokolow-Lyon voltage

In ribociclib-treated breast cancer patients, ECG parameters exhibited significant time-dependent changes (Fig. 4). LME models were adjusted for visit type (D0, D14 ± 3, D28 ± 3), methods (TCM vs. TAM), readers (A vs. B), readings (reading #1 vs. #2), and age. The variables "methods", "readers", and "readings" did not show significant effects (Table S1). By day 14 (vs. D0), HR decreased slightly (−3 ± 1 bpm, *p* < 0.001), QTc increased notably (16 ± 1 ms, *p* < 0.001), QRS duration shortened significantly (−2 ± 0 ms, *p* < 0.01), and the Sokolow voltage using lead V5 rose significantly (47 ± 18 µV, *p* < 0.05), with similar trends using lead V6. By day 28 (vs. D0), QTc decreased (−6 ± 1 ms, *p* < 0.001), returning below baseline values, while the PR interval showed a modest increase (3 ± 1 ms, *p* < 0.05). Both HR, Sokolow voltage and QRS duration also returned to baseline levels (see Fig. 4 and Table S1).

**Fig. 5:**
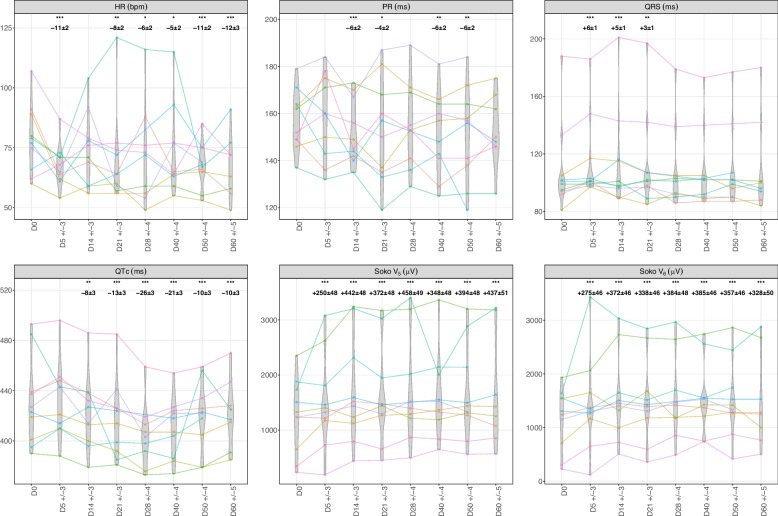
Electrocardiographic changes over time in ICI myocarditis patients. Soko for Sokolow-Lyon voltage

In severe ICI myocarditis patients treated by abatacept, ruxolitinib and corticosteroids, ECG parameters exhibited distinct temporal patterns (Fig. 5). LME models were adjusted for visit type (D0, and 7 other visits between D5 ± 3 to D60 ± 5), methods (TCM vs. TAM), readers (A vs. B), readings (reading #1 vs. #2), age, and sex. The variables "methods", "readers", and "readings" did not show significant effects (Table S2). By day 5 (vs. D0), HR declined significantly (−11 ± 2 bpm, *p* < 0.001) and QRS duration increased (6 ± 1 ms, *p* < 0.001). The HR reduction persisted through day 60 (−12 ± 3 bpm, *p* < 0.001). The PR interval showed modest reductions at day 14 (−6 ± 2 ms, *p* < 0.001) and day 50 (−6 ± 2 ms, *p* < 0.01). From day 21 through day 60, QTc decreased significantly, with the largest reduction observed by day 28 (−26 ± 3 ms, *p* < 0.001), and smaller yet significant decreases persisted through day 60 (−10 ± 3 ms, *p* < 0.001). From day 5 through day 60, QRS duration (increased at day 5: 6 ± 1 ms, *p* < 0.001) returned to baseline values starting day 28, while the Sokolow-Lyon voltage using lead V5 increased significantly (442 ± 48 µV, *p* < 0.001) by day 5, with the maximum increase observed by day 28 (458 ± 49 µV, *p* < 0.001). This latter elevation persisted throughout the follow-up period (see Fig. 5 and Table S2).

## Discussion

This study evaluated the inter- and intra-reader variability of TCM for ECG measurements and compared it to the traditional TAM across critical ECG intervals in two distinct real-life standardized cardio-oncology patient populations. Our findings demonstrate that TCM provides high reproducibility for key ECG parameters, including HR, PR interval, QRS duration, QTc, and Sokolow voltages using V5 and V6, with minimal bias and narrow limits of agreement. These results underscore the robustness and reliability of TCM, as they align with previous studies reporting comparable LOA values for key ECG parameters, such as QTc measured in dedicated Thorough QT trials [18, 19, 25–27]. In practice, CalECG4.2® is even more accurate, due to its semi-automated QRS onset analysis, which improved upon the previous version that required manual QRS positioning [13].

Semi-automated techniques, such as the TCM, offer superior precision compared to traditional manual measurements, with a reported inter-reader variability for QTc measurements ranging in terms of SD from 6 ms for semi-automated methods to 15 ms for manual methods [28, 29]. In comparison, our study showed intra and inter-reader variability below 3 ms, demonstrating a level of precision that aligns with the highest reported accuracies. Such accuracy level of QTc interval measurement is crucial to easily identify drug-induced QT prolongation in real-life setting, and moreover to decrease the numbers of required subjects needed to identify QT-prolongation induced by drugs in clinical trials [18, 27].

CalECG 4.2® uses classical vector-magnitude analysis with deterministic fiducial detection. In our cohort, TCM achieved sub-5 ms repeatability for QTc with tight agreement to TAM. By contrast, many deep-learning ECG systems are trained on human-labelled intervals and can inherit label and pre-processing variability. For example, Bos et al. reported QTc measurement errors up to ~ 10 ms in concealed long QT syndrome detection, [30] and Giudicessi et al. described QTc measurement variability with SD ~ 5–15 ms [31]. These reports highlight that, for absolute interval quantification, current AI implementations may still exhibit non-trivial variability. CalECG 4.2 is a transparent, deterministic alternative that can complement AI approaches. Head-to-head benchmarking on identical raw ECGs with matched filtering and adjudication is needed to establish comparative precision and to determine whether deep learning adds value for complex tasks—such as arrhythmia prediction—beyond QTc measurement [32].

The integration of LME modelling in this study provided a detailed assessment of time-dependent variations in ECG parameters in response to cardiotoxic treatments. Our results highlight a significant temporal effect of ribociclib on QTc, PR, QRS, and Sokolow-Lyon indices, underscoring the dynamic nature of treatment-induced cardiotoxicity. QTc significantly increased by day 14 and shortened back to baseline by day 28, reflecting the transient impact of ribociclib on ventricular repolarization. This pattern aligns with ribociclib's administration schedule, which involves a 21-day treatment cycle followed by a 7-day break [33, 34]. Given its plasma half-life ranging from 29.7 to 54.7 h, higher circulating drug levels are expected during the treatment phase, contributing to the observed ECG changes. Additionally, the PR interval prolongation observed by day 28 indicates a possible atrioventricular conduction delay induced by ribociclib, independent of concentration, unlike its well-documented QT prolonging effect [27, 35]. PR interval prolongation may be less reversible and warrants further evaluation, particularly regarding its potential impact on clinical conduction disturbances. Our study uniquely expands the understanding of ribociclib’s electrophysiological effects by highlighting its possible novel influence on the PR interval, which has not been previously emphasized. However, this relationship requires further clinical evaluations to be fully understood. We also observed a significant increase in Sokolow-Lyon voltage by day 14 on ribociclib. By day 28, the voltage returned near its baseline level. The significance of these changes is unclear, but may suggest a broader electrophysiological impact of ribociclib beyond the known QTc prolongation. This highlights the need for comprehensive assessments in future studies to fully understand its effects on cardiac electrophysiology.

Historically, Sokolow-Lyon voltages have been used to assess left ventricular hypertrophy, although their usefulness has been limited by variability in manual measurements [23]. Nevertheless, when compared to cardiac magnetic resonance imaging or echocardiography, which provide more precise measurements of left ventricular hypertrophy, Sokolow-Lyon voltages often show some level of concordance in identifying significant hypertrophy [36]. In our dataset, the TAM–TCM mean difference for Sokolow–Lyon voltage was ~ 0.01 mV (10 µV) with limits of agreement around − 0.04 to + 0.06 mV—well under 2% of the conventional 3.5 mV threshold for defining left ventricular hypertrophy [36]. This magnitude is unlikely to change hypertrophy classification, even near the cutoff, supporting the practical interchangeability of TCM and TAM for voltage-based screening and serial monitoring. Recent studies suggest that Sokolow-Lyon voltages may also be a marker of proper left intraventricular conduction as it represent the summation of action potentials occurring simultaneously [12]. In cases of myocardial toxicity, particularly those mediated by lymphocytes and macrophages inflammation, the conduction can become disorganized, leading to a lower widespread QRS voltage. This phenomenon is observed in ICI-myocarditis, where the initial disruption in conduction results in a low Sokolow-Lyon voltage [10, 11, 37]. The identification of its improvement under immunosuppressive treatment in this work is novel and highlights its potential as a recovery marker. This perspective shifts the focus from hypertrophy to a broader assessment of electrophysiological integrity.

**Fig. 6:**
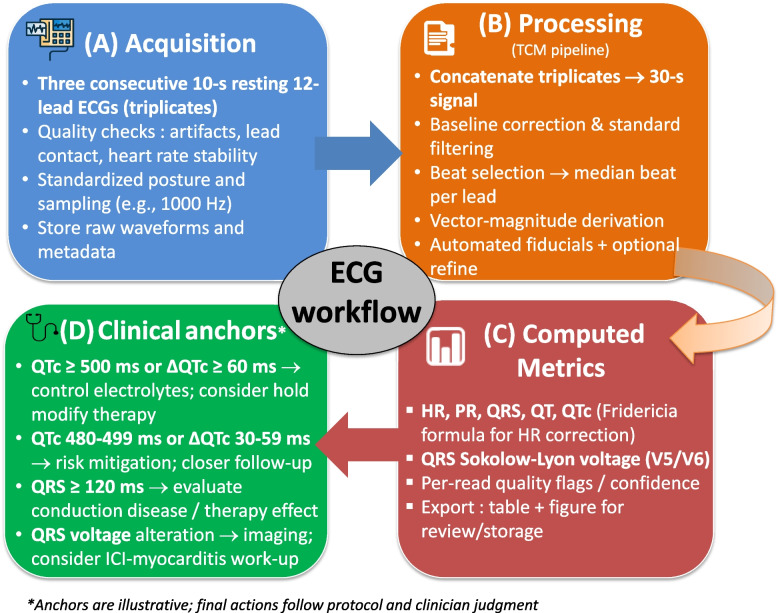
Proposed ECG workflow pipeline from acquisition to clinical decision. Abbreviations: 10-s: 10 s; HR: heart rate; ms: millisecond; QTc: QT corrected for heart rate; TCM: triplicate concatenation method; ΔQTc: QTc variation between baseline pre-drug and after drug intake

To facilitate implementation, the TCM pipeline from acquisition to interval and voltage outputs is summarized (Fig. 6), which also illustrates common decision anchors used in cardio-oncology monitoring [4, 38].

### Limitations

This study has several limitations. Since our analysis focused on two specific patient cohorts, it may not fully capture the variability present in other clinical settings. Variability tends to be lower among healthy volunteers without underlying pathology compared to patients with cardiac conditions, as the latter group presents more complex and variable ECG patterns. This study specifically focuses on patients with cardiac conditions to validate the method's robustness in a highly variable context. Additionally, although ECG analysis is widely used in clinical practice, it is important to acknowledge that the variability in measurements can be influenced by the quality of the ECG waveform. The near-perfect QRS complexes and overall waveform obtained in this study may contribute to the narrow variability, which might not be replicated in all clinical care settings where non-standardized ECG may be performed by less trained workers. All ECGs were recorded on a single platform (Mortara® ELI-280, 1000 Hz sampling, 150 Hz low-pass filter), under standardized acquisition by trained staff. Results (bias/LOA, repeatability) may vary with front-end filtering, sampling rate, gain, lead wire geometry, and electrode placement on other systems; generalizability therefore requires prospective cross-device validation (e.g., Philips, Schiller) and a noise-stress test in less-controlled settings (post-operative/intensive care unit telemetry, ambulatory motion) with controlled artifacts (baseline wander, muscle noise, 50/60 Hz electrical interference, poor contact) and lower sampling (250–500 Hz) sensitivity analyses. These studies will help confirm that TCM performance is robust across hardware and real-world noise conditions. Additionally, this study primarily serves as a validation for our ECG measurement method, and we were unable to integrate comprehensive clinical data and concomitant treatments that could potentially alter ECG parameters beyond the few demographic variables considered in the pathological settings used herein. Future research should aim to incorporate these factors to provide a more complete evaluation.

## Conclusion

TCM demonstrates high reproducibility and minimal variability, establishing itself as a reliable alternative to TAM for ECG interval measurements. Its ability to capture time-dependent changes in ECG parameters, such as QTc, PR, QRS, and Sokolow-Lyon voltages, highlights its potential for monitoring drug-induced cardiotoxicity in cardio-oncology.

## Supplementary Information

Supplementary Material 1: Table S1. Fixed Effects of ECG parameters in Breast Cancer Patients Treated with Ribociclib. The TCM was compared with the TAM, and Reader B was compared with Reader A. "Readings" refers to the comparison between TCM reading#1 and #2. For the intercept, the reference day is D0

Supplementary Material 2: Table S2. Fixed Effects of ECG parameters in severe ICI myocarditis patients. The TCM was compared to the TAM, and Reader B was compared to Reader A. "Readings" refers to the comparison between TCM reading#1 and #2. For the intercept, the reference day is D0

## Data Availability

The data supporting the findings of this study are available from the corresponding author upon reasonable request.

## Abbreviations

ECG: Electrocardiogram
HR: Heart rate
ICC: Intraclass correlation coefficient
ICI: Immune checkpoint inhibitor
LOA: Limits of agreement
LME: Linear mixed-effects modelling
PR: PR interval
QTc: Corrected QT interval (using Fridericia’s formula)
QRS: QRS complex duration
SD: Standard deviation
TAM (Triplicate assessment method): A standard method where three separate 10 s ECGs are recorded and averaged to reduce beat-to-beat variability
TCM (Triplicate concatenation method): Semi-automated method where three 10 s ECG tracings are merged into a single waveform
