# Supplementary material for: Validation of a novel semi-automated ECG quantification tool, applied to a cardio-oncology: Semi-automated ECG Tool applied to cardio-oncology
Source: Cardiooncology. 2025 Dec 19;11:113. doi: 10.1186/s40959-025-00405-7 (PMC12717738; doi:10.1186/s40959-025-00405-7)
Supplement: Supplementary file 1 — Supplementary Material 1: Table S1. Fixed Effects of ECG parameters in Breast Cancer Patients Treated with Ribociclib. The TCM was compared with the TAM, and Reader B was compared with Reader A. "Readings" refers to the comparison between TCM reading#1 and #2. For the intercept, the reference day is D0 [file 40959_2025_405_MOESM1_ESM.docx]

**Table S1.**

**Fixed Effects of ECG parameters in Breast Cancer Patients Treated with Ribociclib.** *The TCM was compared to the TAM, and Reader B was compared to Reader A. "Replicate" refers to the comparison between TCM1 and TCM2. For the intercept, the reference day is D0.*

| **Covariable** | **QTc (ms) †** | **HR (bpm) ‡** | **PR (ms) ‡** | **QRS (ms) ‡** | **Soko V5 (µV) ‡** | **Soko V6 (µV) ‡** |
| --- | --- | --- | --- | --- | --- | --- |
| **Intercept** | 399.3 ± 21.6*** | 76.2 ± 10.1 *** | 115.6 ± 27.3 *** | 48.0 ± 20.7 * | 2502.1 ± 669.1 ** | 2387.8 ± 637.1 ** |
| **D14 ±3** | 15.7 ± 1.4 *** | -3.0 ± 0.5 *** | -1.0 ± 1.1 (ns) | -1.5 ± 0.5 ** | 46.8 ± 18.4 * | 40.4 ± 18.5 (ns) |
| **D28 ±3** | -6.2 ± 1.4 *** | 0.3 ± 0.5 (ns) | 3.0 ± 1.1 * | -0.7 ± 0.5 (ns) | 48.4 ± 18.4 * | 17.1 ± 18.5 (ns) |
| **TCM** | 1.5 ± 1.6 (ns) | -0.0 ± 0.6 (ns) | -0.1 ± 1.3 (ns) | 0.1 ± 0.5 (ns) | -6.7 ± 21.2 (ns) | -5.7 ± 21.4 (ns) |
| **Reader B** | -0.3 ± 1.6 (ns) | 0.0 ± 0.6 (ns) | -0.5 ± 1.3 (ns) | -0.9 ± 0.5 (ns) | 0.0 ± 21.2 (ns) | 0.0 ± 21.4 (ns) |
| **Reading #2** | 0.2 ± 1.6 (ns) | 0.0 ± 0.6 (ns) | 0.6 ± 1.3 (ns) | 1.1 ± 0.5 (ns) | 0.0 ± 21.2 (ns) | 0.0 ± 21.4 (ns) |
| **Age** | 0.4 ± 0.3 (ns) | -0.1 ± 0.2 (ns) | 0.7 ± 0.4 (ns) | 0.8 ± 0.3 * | -12.4 ± 10.5 (ns) | -12.2 ± 10.0 (ns) |

*Soko: Sokolow-Lyon voltage*

**p<0.05, **p<0.01, ***p<0.001, ns = non-significant*, *SD = standard deviation*

† Primary endpoint (QTc): no false discovery rate adjustment (per protocol).
‡ Secondary endpoints (HR, PR, QRS, Sokolow V5/V6): p-values adjusted using Benjamini–Hochberg false discovery rate for the timepoints.
