# Supplementary material for: Validation of a novel semi-automated ECG quantification tool, applied to a cardio-oncology: Semi-automated ECG Tool applied to cardio-oncology
Source: Cardiooncology. 2025 Dec 19;11:113. doi: 10.1186/s40959-025-00405-7 (PMC12717738; doi:10.1186/s40959-025-00405-7)
Supplement: Supplementary file 2 — Supplementary Material 2: Table S2. Fixed Effects of ECG parameters in severe ICI myocarditis patients. The TCM was compared to the TAM, and Reader B was compared to Reader A. "Readings" refers to the comparison between TCM reading#1 and #2. For the intercept, the reference day is D0 [file 40959_2025_405_MOESM2_ESM.docx]

**Table S2.**

**Fixed Effects of ECG parameters in severe ICI myocarditis patients.** *The TCM was compared to the TAM, and Reader B was compared to Reader A. "Replicate" refers to the comparison between TCM1 and TCM2. For the intercept, the reference day is D0.*

| **Covariable** | **QTc (ms) †** | **HR (bpm) ‡** | **PR (ms) ‡** | **QRS (ms) ‡** | **Soko V5 (µV) ‡** | **Soko V6 (µV) ‡** |
| --- | --- | --- | --- | --- | --- | --- |
| **Intercept** | 425.3 ± 104.1 ** | 131.8 ± 3.8 * | 108.3 ± 59.2 (ns) | -45.2 ± 104.6 (ns) | 5302.1 ± 2290.6 (ns) | 5348.2 ± 1764.5 * |
| **D5 ±3** | 1.6 ± 2.6 (ns) | -11.0 ± 2.4 *** | 1.9 ± 1.7 (ns) | 6.2 ± 1.1 *** | 249.7 ± 47.6 *** | 275.0 ± 46.4 *** |
| **D14 ±3** | -8.1 ± 2.6 ** | -4.3 ± 2.4 (ns) | -6.1 ± 1.7 *** | 5.4 ± 1.1 *** | 441.5 ± 47.6 *** | 372.0 ± 46.4 *** |
| **D21 ±3** | -13.0 ± 2.6 *** | -8.1 ± 2.4 ** | -4.1 ± 1.7 * | 3.4 ± 1.1 ** | 371.6 ± 47.6 *** | 338.0 ± 46.4 *** |
| **D28 ±4** | -26.1 ± 2.7 *** | -6.3 ± 2.5 * | -2.3 ± 1.8 (ns) | 0.2 ± 1.1 (ns) | 457.7 ± 49.1 *** | 384.3 ± 47.8 *** |
| **D40 ±4** | -21.1 ± 2.6 *** | -5.3 ± 2.4 * | -5.6 ± 1.7 ** | -0.3 ± 1.1 (ns) | 348.1 ± 47.6 *** | 384.6 ± 46.4 *** |
| **D50 ±4** | -10.2 ± 2.6 *** | -10.7 ± 2.4 *** | -5.6 ± 1.7 ** | 0.9 ± 1.1 (ns) | 394.0 ± 47.6 *** | 357.3 ± 46.4 *** |
| **D60 ±5** | -9.8 ± 2.8 *** | -12.1 ± 2.6 *** | -2.3 ± 1.8 (ns) | -0.7 ± 1.1 (ns) | 436.8 ± 50.9 *** | 328.0 ± 49.6 *** |
| **TCM** | 0.9 ± 1.9 (ns) | 0.2 ± 1.7 (ns) | -0.9 ± 1.2 (ns) | -0.5 ± 0.8 (ns) | -13.3 ± 34.3 (ns) | -13.7 ± 33.4 (ns) |
| **Reader B** | 0.4 ± 1.9 (ns) | 0.0 ± 1.7 (ns) | -0.4 ± 1.2 (ns) | -1.2 ± 0.8 (ns) | -0.7 ± 34.3 (ns) | -0.7 ± 33.4 (ns) |
| **Reading #2** | 0.2 ± 1.9 (ns) | 0.0 ± 1.7 (ns) | 0.6 ± 1.2 (ns) | 1.3 ± 0.8 (ns) | 0.5 ± 34.3 (ns) | 0.3 ± 33.4 (ns) |
| **Age** | 0.1 ± 1.3 (ns) | -0.7 ± 0.5 (ns) | 0.6 ± 0.7 (ns) | 2.0 ± 1.3 * | -58.4 ± 28.5 (ns) | -58.9 ± 21.9 * |
| **Sex (Male)** | -7.5 ± 22.6 (ns) | -5.4 ± 8.3 (ns) | 3.9 ± 12.9 (ns) | 11.4 ± 22.7 (ns) | 392.7 ± 497.5 (ns) | 256.6 ± 383.2 (ns) |

*Soko: Sokolow-Lyon voltage*

**p<0.05, **p<0.01, ***p<0.001, ns = non-significant, SD = standard deviation*

† Primary endpoint (QTc): no false discovery rate adjustment (per protocol).

‡ Secondary endpoints (HR, PR, QRS, Sokolow V5/V6): p-values adjusted using Benjamini–Hochberg false discovery rate for the timepoints.
